# Electroencephalographic evaluation of acoustic therapies for the treatment of chronic and refractory tinnitus

**DOI:** 10.1186/s12901-017-0042-z

**Published:** 2017-11-28

**Authors:** Luz María Alonso-Valerdi, David I. Ibarra-Zarate, Francisco J. Tavira-Sánchez, Ricardo A. Ramírez-Mendoza, Manuel Recuero

**Affiliations:** 1 Escuela de Ingeniería y Ciencias, Tecnológico de Monterrey, Eugenio Garza Sada 2501, 64849 Monterrey, NL Mexico; 2 Grupo de Investigación en Instrumentación y Acústica Aplicada (I2A2), Universidad Politécnica de Madrid, Carretera de Valencia km 7, 28031 Madrid, Spain; 3 Massachusetts Institute of Technology, Cambridge, MA USA

**Keywords:** Tinnitus, Acoustic therapy, Electroencephalography (EEG), Auditory perception, Neural oscillations

## Abstract

**Background:**

To date, a large number of acoustic therapies have been applied to treat tinnitus. The effect that produces those auditory stimuli is, however, not well understood yet. Furthermore, the conventional clinical protocol is based on a trial-error procedure, and there is not a formal and adequate treatment follow-up. At present, the only way to evaluate acoustic therapies is by means of subjective methods such as analog visual scale and ad-hoc questionnaires.

**Methods:**

This protocol seeks to establish an objective methodology to treat tinnitus with acoustic therapies based on electroencephalographic (EEG) activity evaluation. On the hypothesis that acoustic therapies should produce perceptual and cognitive changes at a cortical level, it is proposed to examine neural electrical activity of patients suffering from refractory and chronic tinnitus in four different stages: at the beginning of the experiment, at one week of treatment, at five weeks of treatment, and at eight weeks of treatment. Four of the most efficient acoustic therapies found at the moment are considered: retraining, auditory discrimination, enriched acoustic environment, and binaural.

**Discussion:**

EEG has become a standard brain imaging tool to quantify and qualify neural oscillations, which are basically spatial, temporal, and spectral patterns associated with particular perceptual, cognitive, motor and emotional processes. Neural oscillations have been traditionally studied on the basis of event-related experiments, where time-locked and phase-locked responses (i.e., event-related potentials) along with time-locked but not necessary phase-locked responses (i.e., event-related (de) synchronization) have been essentially estimated. Both potentials and levels of synchronization related to auditory stimuli are herein proposed to assess the effect of acoustic therapies.

**Trial registration:**

Registration Number: ISRCTN14553550. ISRCTN Registry: BioMed Central. Date of Registration: October 31st, 2017.

## Background

**Fig. 1:**
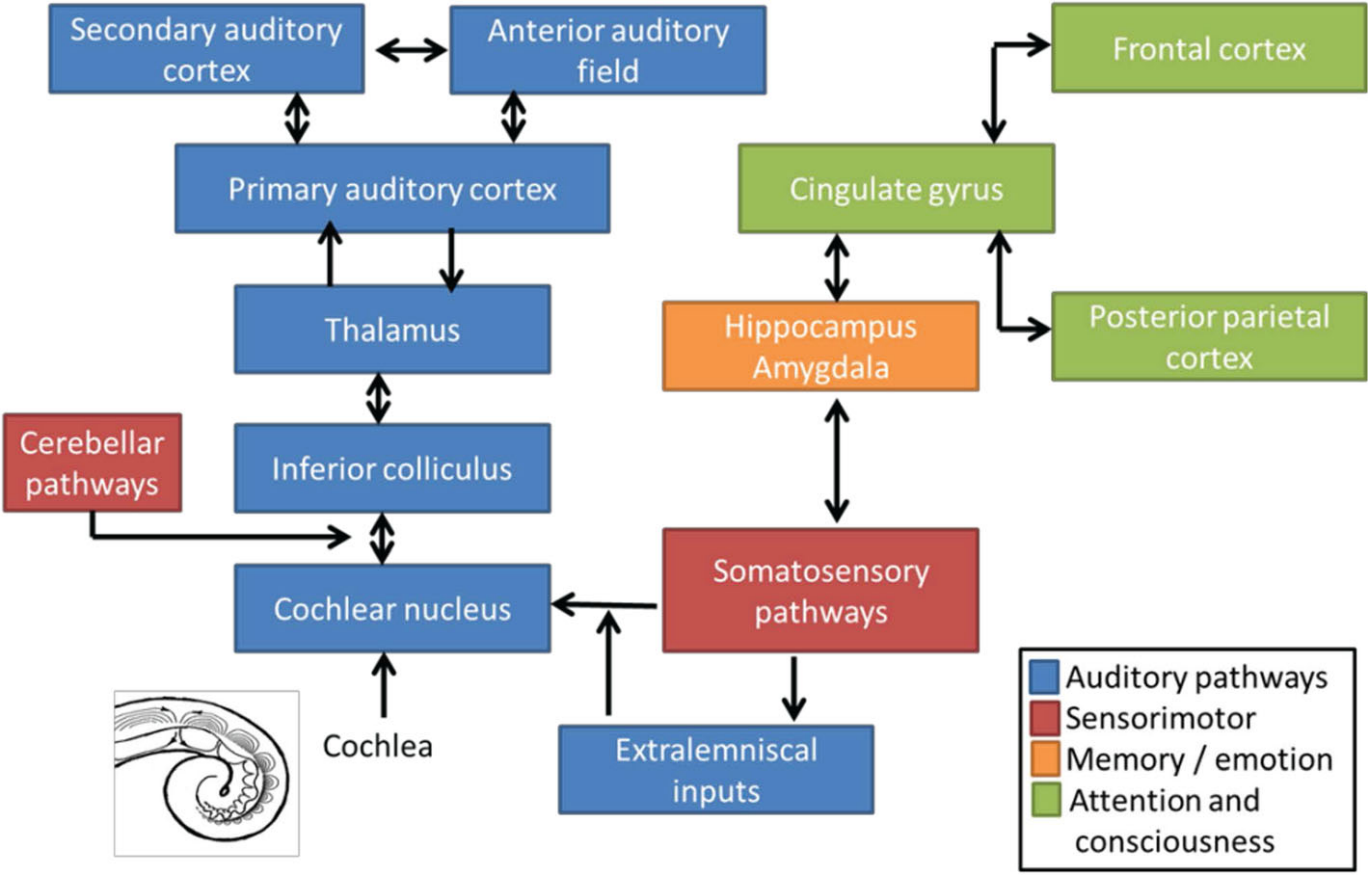
Simplified representation of auditory and non-auditory pathways in tinnitus

The auditory system is aimed at hearing and balance. It is constituted by the peripheral hearing system (outer, middle and inner ear) and the central auditory system (primary and association cortices), both of them interconnected via the auditory nerve. Overall, the hearing process takes place as follows. First, air acoustic signals are converted to mechanical vibrations in the peripheral hearing system. Then, mechanical vibrations are transduced into electric signals within the cochlea. Finally, electrical signals are conducted through the auditory nerve to the auditory cortex, where they are decoded. As more complex the auditory stimulus is, auditory cortices will be more involved in the processing of information, particularly the association cortex [1–3]. In this respect, auditory neural processing takes place initially within the cochlear nucleus. Afterwards, it moves forward from inferior colliculus to thalamus and auditory cortex. Auditory processing outputs expand to several major non-auditory neural areas, including those associated with memory, emotions, attention, consciousness and sensorimotor processing [4]. Refer to Fig. 1.

An auditory problem that affects 5–15% of the world population is ***tinnitus*** [5]. In 1% of the cases, it may be considered as impairment which affects the daily life [6, 7]. Tinnitus is an auditory phantom percept of chronic high pitched sound, typically in the frequency range of 6-8 kHz, without any external sound source [8, 9]. Tinnitus percept is usually simple, with common forms resembling pure tones (‘ringing’), Gaussian noise (‘hissing’), or buzzing [10]. In 1982, the Committee on Hearing, Bioacoustics and Biomechanics of the United States of America defined tinnitus as a conscious experience of a sound that is originated from patients’ own head. Later, in [11], it was redefined the term as the perception of a sound that results exclusively from nervous system activity, and without any corresponding mechanical vibratory activity proceeding from the cochlea. Additionally, in [12], tinnitus was classified into objective and subjective. Objective tinnitus (somatosensory sounds) was associated with peripheral vascular abnormalities detectable by stethoscopic inspection, whereas subjective tinnitus was determined as an acoustic perception merely experienced by the patient. In line with acoustic principles, tinnitus is assumed subjective and is defined in terms of loudness and pitch. To characterize tinnitus is necessary a frequency response curve that is obtained objectively in animals [13], but subjectively in human beings [14].

Tinnitus may be caused by exposure to loud noise, fever, ototoxicity or a transient disturbance in the middle ear. It eventually disappears in most of the cases, but it remains in 15% of the affected population [15]. Tinnitus can be perceived by people of all ages, either those with normal hearing or those with deafness. Interestingly, when a hearing loss exists, the pitch of tinnitus corresponds to the frequency region of that hearing loss [16]. The probability of a person to suffer from tinnitus increases with age and degree of hearing loss: around 12% of patients suffering from tinnitus are over 60 years, while only 3% of them are between 20 and 30 years [17]. In 3% of the total affected population, tinnitus is enough intense to deteriorate the quality of life. Tinnitus commonly provokes sleep disturbance, restricted working capacity, difficulties on attention, psychiatric anguish, anxiety and depression [15, 18]. According to [11], people experiencing tinnitus can be divided into five main categories: (i) *Category 0*, patients without hyperacusis or hearing loss, and whose tinnitus has little impact on their daily lives; (ii) *Category 1*, patients with a significant tinnitus but without hiperacusia or hearing loss; (iii) *Category 2*, patients with both significant tinnitus and hearing loss; (iv) *Category 3*, patients experiencing significant hyperacusis for a long period of time with or without tinnitus; and (v) *Category 4*, patients with progressive tinnitus and hyperacusis.

The origin of tinnitus is still unknown. Tinnitus is generally triggered by hearing loss, and very often by noise-induced hearing loss, but most chronic tinnitus is of central origin; that is, it is in the brain and not generated in the ear. Head or neck injuries can also trigger tinnitus by altering somatosensory inputs, which in turn affect auditory pathways and lead to tinnitus or modulate its intensity. Emotional and attentional state could be also involved in the development and maintenance of tinnitus via top-down mechanisms [19]. Neuroimaging studies in humans and animals suggest that tinnitus is associated with increased neural synchrony, reorganization of tonotopic maps, and increased spontaneous firing rates in the auditory system [16]. Specifically, electrophysiological studies have revealed that the brain oscillatory activity decreases in the alpha band (10–14 Hz), and increases in delta (1.5–4 Hz) and gamma (> 30 Hz) bands [20]. Abnormal brain oscillations on the frontal lobe have been found as well. As this lobe is related to the emotional and attentional regulation, frontal irregularities have been associated with stress caused by tinnitus [21]. On the other hand, a number of functional brain imaging studies has also shown aberrant neural activity within the central auditory pathway of tinnitus patients. Changes in the inferior colliculus, the thalamus and the auditory cortex have been demonstrated by using both Positron Emission Tomography [22–30] and functional Magnetic Resonance Imaging [31–33]. Alterations of neural activity were also observed in non-auditory brain structures, especially in the limbic system [29, 34, 35]. Using high-resolution magnetic resonance imaging and voxel-based morphometry, circumscribed alterations in the auditory system (medial geniculate nucleus of the thalamus) and the limbic system (subcallosal region including the nucleus accumbens) were detected [36].

To date, there is no medical, neurological, or neurophysiological therapy that has been proved to cure tinnitus [5]. Therefore, there is a wide variety of treatments for tinnitus, including hearing aids, maskers, counseling, retraining therapy, music therapy, acupuncture, herbal treatments, and acoustic therapy [37]. Particularly, acoustic therapies aim to reverse the neuroplasticity phenomenon related to tinnitus by adequately stimulating the auditory pathway. If neuroplastic changes are produced, habituation and/or suppression of the tinnitus may be achieved [38]. Habituation refers to the elimination of the tinnitus effects without eliminating sound perception per se. In contrast, suppression is the reduction or disappearance of the tinnitus perception. The suppression might last from a few seconds to days. Some of the most relevant acoustic therapies so far are the following: tinnitus masking therapy [12], tinnitus retraining therapy [11], tinnitus phase-out [39–41], high frequency therapy [42, 43], auditory discrimination therapy [44, 45], therapy for enriched acoustic environment [20], binaural therapy [46], and neuromodulation [21, 47].

Even though a large number of acoustic therapies have been designed to treat tinnitus, the effect that produces the corresponding auditory stimulus is not well understood yet. Up to now, the effectiveness of the acoustic therapies to treat patients suffering from tinnitus is evaluated by means of a visual analog scale and/or ad hoc questionnaires. The visual analog scale is used to quantify subjectively certain sensations of the patients such as pain. This scale is a line whose ends respectively mean lack of sensation and extreme feeling. What patients do is to mark a point on the line that matches their sensation magnitude. On the other hand, the questionnaires consist of a series of questions that allow identifying the difficulties that patients are facing due to the tinnitus, and whether those difficulties are overcome after an acoustic therapy had been applied. Typically, only three responses are provided in the questionnaires: yes, sometimes, and no [20, 21]. Despite the popularity of these two methods, the resulting evaluation is completely subjective and does not allow an effective quantification of the acoustic therapy effects. This has leaded to apply the acoustic therapies following a trial-error procedure, what delays the patient healing, or even could deteriorate the patient condition.

As very little attention has been paid to the importance of an objective method to evaluate acoustic therapies used to treat tinnitus [21, 47], this research protocol seeks to establish a new methodology based on the treatment monitoring at a cortical level. Since a likely cause of tinnitus is the neuronal hyperactivity in the nervous system, EEG which measures non-invasively the electrical activity of the cerebral cortex seems to be a feasible method to assess objectively the acoustic therapy effects. Having in mind all the issues raised herein, there are two primary aims of this study: (1) To apply four (retraining, auditory discrimination, enriched acoustic environment, and binaural) of the most successful acoustic therapies at present to patients suffering from tinnitus, and who had received other palliative treatments with no positive results; and (2) to record the EEG signals of those patients before, during and after the application of the corresponding acoustic therapy, so as to analyze the neural behavior of auditory and non-auditory nervous systems. The study of spontaneous activity at resting state, evoked activity and induced activity will be undertaken after data collection.

As the goal of the acoustic therapies is the habituation or suppression of tinnitus, it is hypothesized that these therapies should produce perceptual and cognitive changes (specifically those related to attention and memory), even though they could not be beneficial. It is expected that the patients’ EEG signals along the acoustic therapy will reveal neural modifications, which could explain why this treatment is so effective in some cases, and waste of time in some others. Results from this research might help to pointing out acoustic therapies as a potential solution for certain patients, but not a viable treatment for many others. Tinnitus has been proposed as an abnormal activity proceeding from multifunctional neural networks [48], and its heterogeneity hinders to find a universal cure to treat it. As a result, to establish an objective methodology which could approve or discard acoustic therapies as a feasible tinnitus treatment is a worthwhile research.

The present protocol was approved by the Ethical Committee of the Tecnológico de Monterrey (CONBIOETICA19CEI00820130520) on June 20th 2016, and has been recently attracted L’Oréal-UNESCO Organization as a sponsor. The research project is undertaken in collaboration with the National Institute of Rehabilitation (Mexico City), where patients with chronic and refractory tinnitus interested in being treated with acoustic therapies are recruited for the study. Materials, equipment, and procedures are specified in the remaining part of this document.

## Methods

### Generation of the acoustic therapies

In order to generate the acoustic therapies, two main software programs are used: MATLAB and Audacity. MATLAB is a proprietary programming language developed by MathWorks, whose software license is available in the Tecnológico de Monterrey. Audacity is free open-source audio software for multi-track recording and editing [49]. The acoustic therapies are designed in line with the patient audiology evaluation carried out by the National Institute of Rehabilitation. Such evaluation includes audiometry, hearing loss, tinnitus pitch matching and tinnitus handicap inventory. The main adjustment parameters are taken from the audiometry, which measures the ability of each ear to perceive the vibrations within different frequency bands of the audible spectrum.

As was stated before, four of the eight acoustic therapies herein described are considered. Patients suffering from chronic and refractory tinnitus coming from the National Institute of Rehabilitation are submitted to a random selection so as to belong to a therapy group or a control group. The acoustic stimuli for each group is specified in the forthcoming sections [50–53].

#### Control group

For this group, a simple relaxing music is employed and the same rules followed in the therapy groups are established.

#### ADT group

**Fig. 2:**
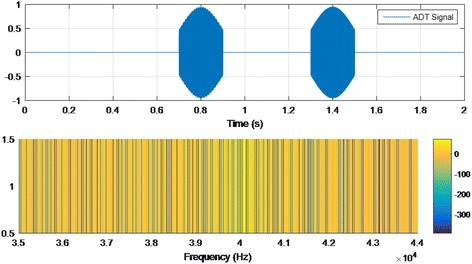
Sample of ADT signal: time and frequency analysis

Auditory discrimination therapy (ADT) requires the attention of the patient on the therapy. The vast majority of published works on ADT use oddball paradigms as stimulus. These paradigms consist of composed sound of standard and deviant pulses, presented in a random way. The patient has to note which type of pulse is involved (standard or deviant). The standard pulse is white noise with duration of 500 ms (50% probability). The deviant pulse can range from 4 to 8 kHz with duration between 50 and 100 ms (50% probability). The inter-latency between pulses could be around 1.5 s. A sample test of ADT signal is shown in Fig. 2.

#### TRT group

**Fig. 3:**
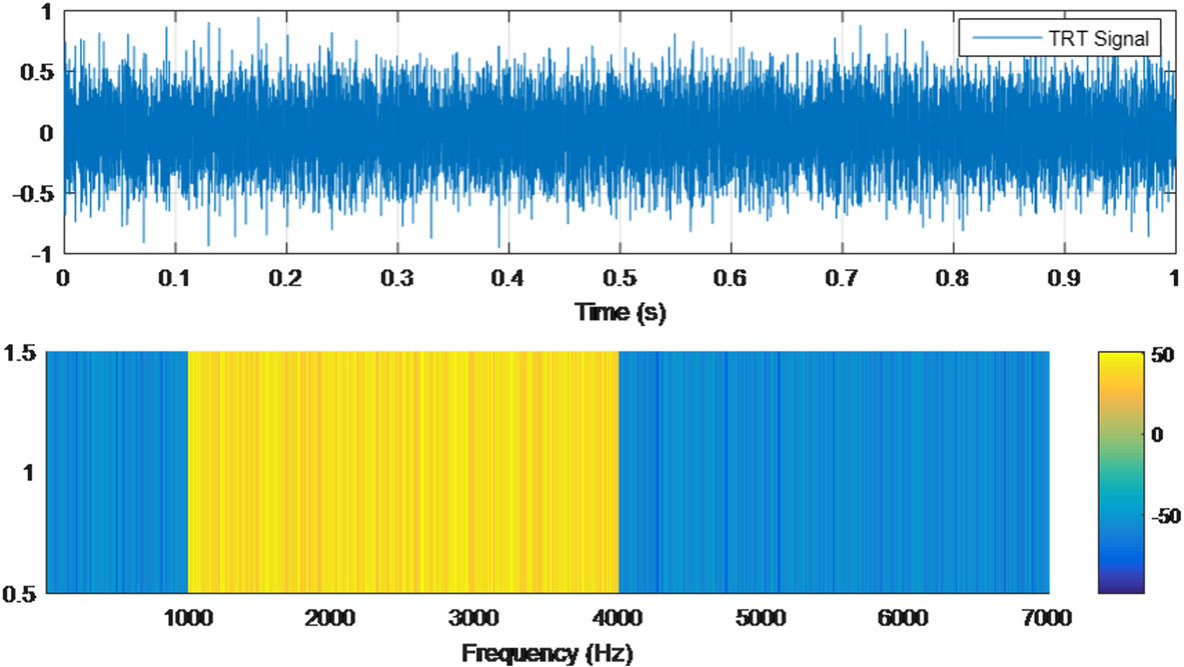
Sample of TRT signal: time and frequency analysis

To generate tinnitus retraining therapy (TRT), a random noise signal is used. This signal is additionally filtered by octave bands, depending on the tinnitus frequencies and hearing loss in each ear. This therapy has two main objectives: to get used to the reactions of limbic-autonomic systems, as well as to the tinnitus perception. TRT can be effective, regardless of the etiology of tinnitus. TRT is achieved by directive counseling and exposure to low-level broadband noise. The first component of TRT, directive counseling, may change the way tinnitus is perceived. The patient is taught the basic knowledge about the auditory system and its function, the mechanism of tinnitus generation and the annoyance associated with tinnitus. The repetition of these points in the follow-ups helps the patient to perceive the signal as a non-danger. The second element of TRT therapy, sound therapy, aims to decrease the sound contrast between tinnitus and silent environment leading to a reduced detection of tinnitus. A sample test of TRT signal is shown in Fig. 3.

#### EAE group

**Fig. 4:**
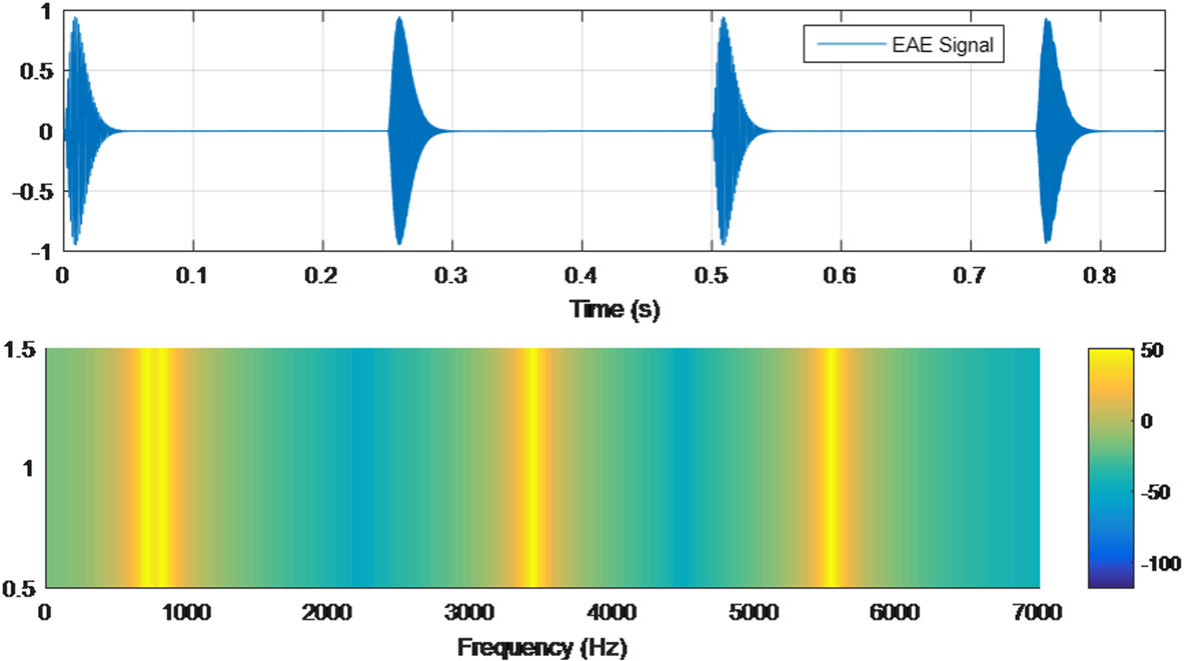
Sample of EAE signal: time and frequency analysis

Therapy for enriched acoustic environment (EAE) is based on a sequence of random frequency tones (burst and pip pulses) with amplitude proportional to the hearing lost reported on patients’ audiometry. Frequency pulses stimulate the auditory pathway in a selective and personalized way. The stimulation is selective, because each tone of the sequence has a response curve in frequencies very similar to the curves of tuning neurons of the auditory pathway [54]. The stimulation is personalized and enriched, because it is designed based on the hearing loss of each patient. A sample test of EAE signal is shown in Fig. 4.

#### Binaural group

**Fig. 5:**
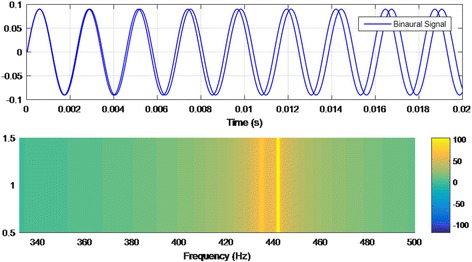
Sample of binaural signal: time and frequency analysis

Psychoacoustics is essentially the study of the perception of sound. This includes how the human listens to, the psychological responses, and the physiological impact of music and sound on the nervous system [55]. Binaural therapy is the one that has more evidence of psychoacoustic effects than the other therapies. The process of reproducing this binaural effect using audio technology was originally developed in the early 1970’s by Gerard Oster, a biophysicist from New York City [56]. A notable example of the binaural effect is the work presented in [57], where neural oscillations and binaural therapy at 10 Hz were synchronized. A sample test of binaural signal is shown in Fig. 5.

### Electroencephalographic evaluation

EEG is a non-invasive and relatively non-expensive technique that measures the electric fields produced by the pyramidal neurons of the cerebral cortex during the synaptic communication. The synapse is the communication bridge between the axon (output) of a neuron and dendrites (inputs) of the next neuron. The number of synaptic inputs (dendrites) that are simultaneously excited determines the amplitude of the EEG signals. EEG signals are also characterized by the frequency at which oscillates the neural networks, and it can range between 0.01 and 600 Hz. In general, perception, cognitive states, and motor activity determine the oscillation frequency of the neural networks [58]. EEG is an extensively used method that gives an insight into the complex and dynamic mechanisms of the human brain: from the decoding of external stimuli registered by the sense organs, to the programming of a reaction executed through the muscles [59].

In tinnitus research, neuroimaging techniques have been used to find the association of this neurological disease with alterations in different brain areas. Neural systems related to attention, perception, distress, memory and emotions have been of special interest. EEG findings are particularly discussed hereinafter, along with their relevance for the present work.

#### Spontaneous activity at resting state

Several theories on the neural anomalies due to tinnitus have been proposed. Most of investigators have opted to examine tinnitus effects at resting state, since the human brain generates spontaneous fluctuations in widely separated brain regions, and within a wide frequency range (0-100 Hz). Traditionally, neurophysiological effects of tinnitus have been assessed under the following conditions: (1) comparison of brain activity between tinnitus and non-tinnitus participants; (2) experiments undertaken in a quiet room; (3) participants in seated position in most of the cases; (4) EEG montage with different number of electrodes, from 19 to 128 recording sites; (5) eyes-close (EC), eyes-open (EO), and thereof conditions have been considered; (6) EEG recordings from 2 to 10 min long have been taken, and (7) power and amplitudes of EEG signals across delta, theta, alpha, beta and gamma frequency bands have been particularly explored [5, 48, 60–65].

The main findings of aforementioned studies have provided neurophysiological and neuro-dynamic evidence to show that tinnitus is due to both bottom-up and top-down dysfunctions. This means that not only peripheral auditory system, but central non-auditory cognitive system is also involved in tinnitus genesis [61]. In terms of the auditory system, EEG synchronization of delta, theta, and beta bands have been detected as indicative of a chronic dysrhythmia of thalamus cortical circuits, following auditory deafferentation [5]. Tinnitus loudness has been associated with enhanced delta and gamma band activity on the contralateral auditory cortex. Gamma activity has been also correlated to perception of tinnitus, level of attention directed towards it, and emotions generated by such perception [65]. With respect to the non-auditory system, tinnitus-related emotional distress has been associated with alpha, beta and gamma frequency bands [5].

#### Evoked activity: Event-related potentials (ERPs)

Event-related potentials (ERPs) are time-locked and phase-locked brain responses associated with particular perceptual, cognitive, motor and emotional states [62, 66–68]. They are of two types: exogenous and endogenous. Exogenous components appear within 100 ms after stimulus onset, and are defined by the physical characteristics of such stimulus (e.g., intensity, tone, frequency, pitch and timbre). Endogenous components depend on psychological variables such as attention or task relevance [69–71]. In particular, auditory ERPs are originated from primary cortical areas and depend on the cognitive state of the patient. Some typical auditory ERPs are P1, N1, P2 and N2, which respectively occur 50, 100, 170 and 250 ms after stimulus onset [71].

N1, P2 and N2 have been used to quantify the effects of tinnitus at a neural level. It has been shown that patients suffering from severe tinnitus have significant changes in amplitude and latency of their ERPs. It has also been observed that the reaction times of these patients are much longer than healthy individuals [72]. For example, in [73], and then in [74], it was found that N1, N2, P2, and P3 components (evoked potential for decision making) of patients with chronic tinnitus showed longer latencies in comparison with those of healthy people. Similarly, a significant decrease in amplitude of the P3 component was found. This could be the result of a deficiency in the central auditory processing system due to tinnitus [73, 74]. Additionally, in [75], it was found that patients experiencing severe tinnitus, and who had been unsuccessfully treated with acoustic therapy, did not reflect any change on either N1 or P2 components. Researchers concluded that no habituation to tinnitus was achieved. Finally, in [76], it was analyzed the N1 component of patients with chronic tinnitus and suggested that their attention was very focused on tinnitus, making difficult the use of acoustic therapies. In general, N1 component has been the most widely used and robust response to assess auditory brain function in cerebrovascular diseases, schizophrenia and tinnitus [77].

#### Induced activity

Another way to analyze neural processing is the quantification of the level of (de) synchronization of the EEG signals. This technique is called event-related oscillations (EROs) and what reflects is the neural processing of internal (e.g., level of attention) and external (e.g., motor activity) events. EROs appear in specific bandwidths and both processes (synchronization and desynchronization) may exist simultaneously [78]. EROs can be analyzed in time by mapping the event-related desynchronization (ERD) and event-related synchronization (ERS) and in frequency by determining the event-related spectrum.

ERD/ERS maps allow visualizing the neural processing of the cerebral cortex from a few milliseconds to several seconds. This is a method that was proposed in [78], and which has been significantly enhanced in [79, 80]. In particular, auditory ERD/ERS maps have been mainly investigated in [81–83]. Some of their findings are outlined as follows: (1) Auditory ERD/ERS maps reflect cognitive and attentional processes, rather than the decoding of the auditory stimulus per se; (2) auditory stimulus processing is associated with alpha band synchronization between 10 and 12 Hz; (3) auditory memory increases the level of synchronization of the neurons; (4) the comparison of sounds increases the level of desynchronization of the neurons; and (5) auditory stimuli modify the level of neural synchronization in temporal and parietal lobes.

With regard to the event-related spectrum, this technique allows to study neural dynamics that ERPs and ERD/ERS maps cannot reflect such as phase modifications of the EEG signals (ERP pitfall) and broadband frequency variations (ERD/ERS drawback). Previous studies [84–87] have demonstrated that auditory stimuli evoke neural synchronization around 40 Hz, and that such synchronization increases if the attention focused on the stimulus increases as well.

Even though, ERD/ERS analysis has not been undertaken yet to monitor electrophysiological anomalies of patients suffering from tinnitus, we consider that auditory EROs could be a valuable tool on the basis of present evidence. Similarly, if acoustic therapies are being applied successfully, the effect might be detectable by the same means.

### Analysis strategy

The existing body of research concerning neuroimaging studies on tinnitus has attempted to validate EEG measures as a biomarker to diagnose tinnitus and monitor its progress. Although no validation has been achieved yet, analysis of spontaneous and evoked activity has revealed valuable information. In the case of spontaneous activity examination, modulation of brain rhythms in delta, theta, alpha, beta and gamma frequency bands has been associated with chronic dysrhythmias of thalamus cortical circuits, tinnitus loudness and perception, level of attention, and intensity of destructive emotions. Newly research conducted in [60], it was found no association between psychoacoustic and psychosocial scores and brain oscillatory activity at the time to assess tinnitus perception. They concluded that EEG rhythms should not be considered as a viable biomarker or outcome measure in clinical trial of tinnitus [60]. However, further improvements on analysis procedures followed up to now could lead to obtain more concrete results. This can be illustrated briefly by the work of Klimesch. He considered that theoretical EEG frequency bands were not appropriate since they vary according to age, sex, and current mental state [88]. Furthermore, he recently proposed a new method to calculate real frequency bands in accordance with heart rate [89]. Possibly, the calculation of individual EEG frequency bands for each patient may permit a more properly spontaneous activity examination in our study. Regarding evoked activity analysis, the EEG waveform P1-N1-P2 has been the most studied. The three components are maximal on fronto-central regions and are involved in auditory stimulus processing, attention mechanisms, and memory processes. The vast majority of studies on ERPs [71–76] have found that patients suffering from tinnitus have generally shorter and later components than healthy people, perhaps owing to deficiencies in the central auditory systems. Recently, in [77], it has been questioned the validity of ERP-assessment since the majority of studies only uses 1 kHz tones. These tones have been preferred because they evoke optimal auditory ERPs, but there is no relevance between the auditory stimulus and tinnitus. The selection of relevant auditory stimulus to evoke EEG activity in order to investigate the cortical reorganization due to tinnitus evolution is discussed in Section Passive Mode.

Apart from the examination of spontaneous and evoked EEG activity to evaluate the tinnitus perception, ERD and ERS of EEG signals may be also very helpful, since they reflect important aspects of sensory, motor, and cognitive cortical processing. ERD and ERS have been successfully used to study the neural response in clinical cases such as pain evaluation [90] and neuro-rehabilitation [91]. The enhancement of ERD/ERS quantification must be, however, considered as well. In [92], it was demonstrated that the conventional method proposed in [79] to quantify ERD/ERS introduces a positive bias, resulting in an overestimation of ERS and an underestimation of ERD. Authors proposed to combine single-trial baseline subtraction approach, in conjunction with partial least square regression, to achieve a correct detection and quantification of ERD/ERS [92].

In view of the above argument, the analysis of spontaneous, evoked, and induced EEG activity using improved techniques could lead us to evaluate properly the effectiveness of acoustic therapies to treat tinnitus. In addition, the consideration of other EEG measures such as phase, neural generators, and cross-frequency-coupling might fulfill a broad and thorough assessment of acoustic therapies [93].

### Analysis tools

To undertake the EEG signal processing, two high level programming languages are used: MATLAB and Python. With respect to MATLAB, EEGLAB which is an open-source MATLAB toolbox for processing continuous and event-related EEG signals are essentially employed [94]. As regards Python, *Wyrm* that is a pythonic toolbox for on-line and off-line data analysis might be very useful [95]. Although *Wyrm* was created for brain-computer interfacing, this has a wide variety of tools for processing EEG signals. Note that Python is an open-source programming language, and as efficient as MATLAB.

### Facilities

#### Laboratory

**Fig. 6:**
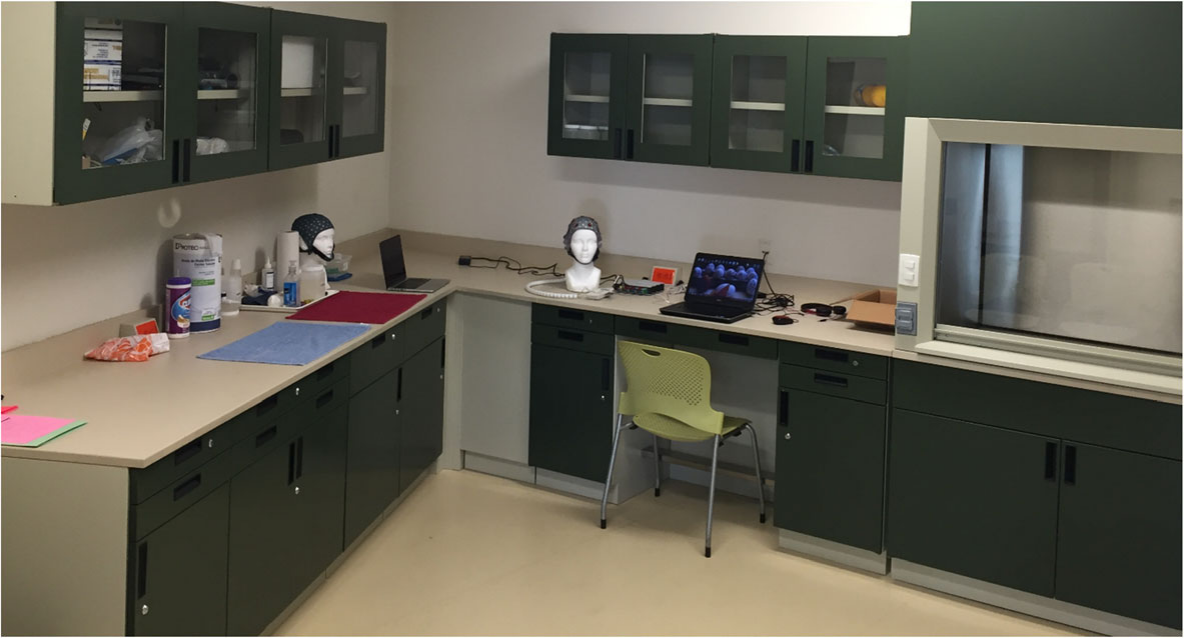
Available laboratory at Tecnológico de Monterrey, where the project herein proposed is run

Patients are examined in a well-equipped research laboratory (Fig. 6) with appropriate conditions to attend people, and record EEG data. Those conditions include a quiet atmosphere, water and toilet services, washing facilities, quiet air conditioner and parking place. In addition, this laboratory was chosen because of the low background noise, which was moreover measured before testing and was around 35 dBA. This parameter is good enough to listen the acoustic therapies and to record EEG data.

#### Equipment and software

**Fig. 7:**
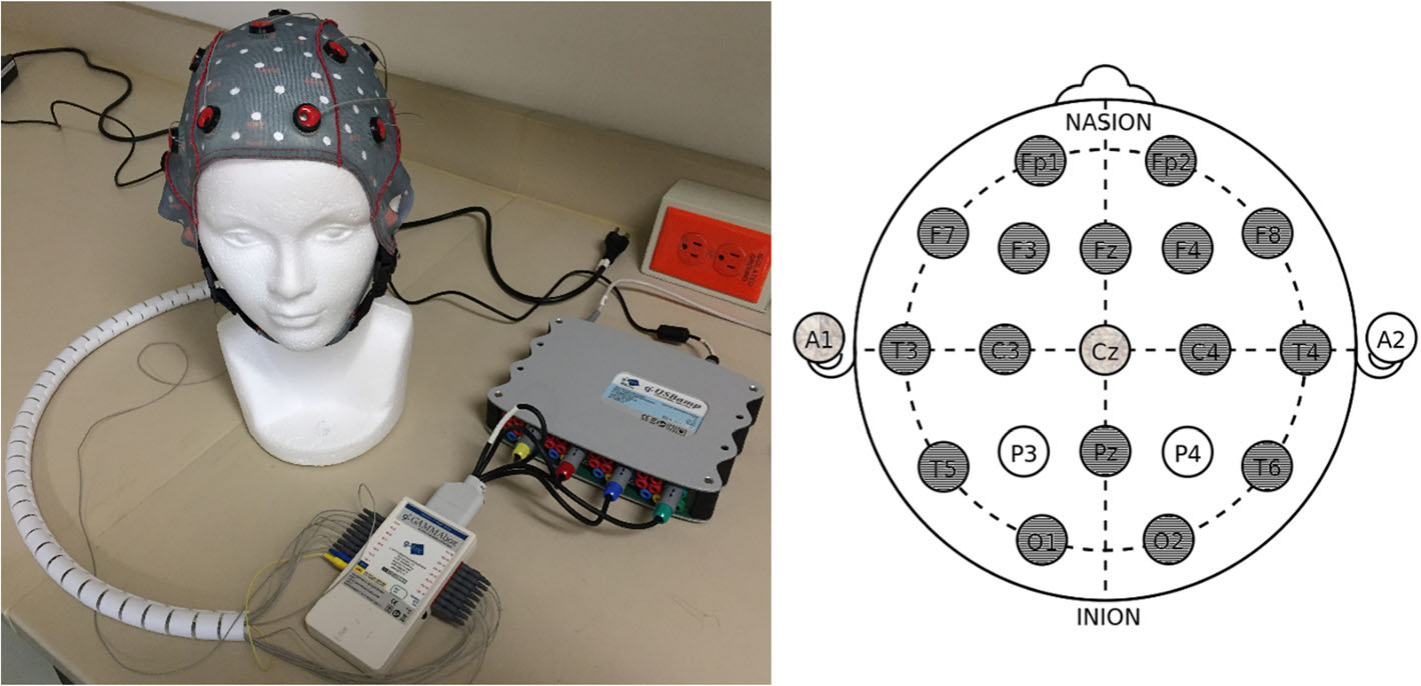
Facilities in the Tecnológico de Monterrey to undertake the project: On the left, the equipment that is employed to record the EEG signals; and on the right, the 17 EEG channels based on the 10/20 system which are used. Only channels P3 and P4 could not be recorded because of infrastructure limitation

To record EEG data, a g.USBamp is available at the laboratory (Fig. 7a). The g.USBamp is a high-performance and high-accuracy biosignal amplifier. It allows acquiring sixteen EEG channels at a sampling frequency of up to 32.7 kHz. The amplifier is configured to sample at 256 Hz within a bandwidth between 0.1 and 100 Hz. The Cz channel is used for referencing the other sixteen EEG channels, and left lobe ear works as ground. This EEG configuration is illustrated in Fig. 7b.

To set-up the experimental procedure, OpenViBE software is utilized [96]. OpenViBE is open-source multi-platform software for brain-computer interfaces and real-time Neurosciences. This software is a very feasible solution since it has an easy to use graphical language, and it also provides a compatible acquisition server with g.USBamp.

#### Devices for acoustic therapies

To end Section Facilities, it is worth noting that audio players are provided to patients. These audio players have a 4Gb memory, and include a pair of headphones and a battery charger. No cost for the audio player is charged.

Owing to the audio chain (player + headphones), it is possible that the acoustic signal that the patient receives through headphones, loses fidelity given the physical and electronic properties of the instruments involved in the audio chain. Therefore, it is necessary to control the audio properties of the sound in the laboratory with the proper equipment. In order to evaluate the audio quality, the audio chain is composed by acoustic signal, audio player, analyzer, headphones and sound pressure level.

**Fig. 8:**
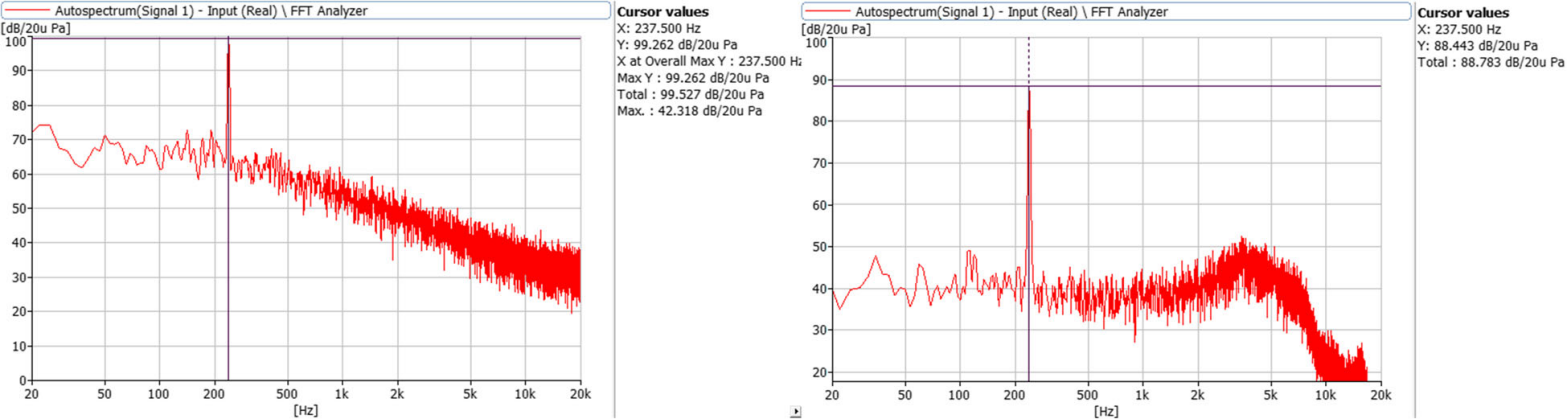
Frequency response of audio player playing a pure tone at 232 Hz

In order to know the error between the design of the acoustic therapy and its application, it was necessary to verify the signal along to the audio chain, and two tests were carried out. First, the output of audio player was connected to the input of the analyzer and the signal was recorded. Figure 8 (on the left) shows the frequency response of a selected frequency. As can be seen from the figure, there is either no distortion or other kind of impurities in the original signal. Second, the sound pressure emitted by the headphones was measured by a Sound Level Meter as a flat wave front, and its frequency response was recorded with a Fast Fourier Transform analyzer (see Fig. 8, right side). There is no distortion in the signal and only a slight increase between 3 and 4 kHz due to intrinsic components of the headphones. This frequency response of the headphones is sufficiently good to be used for acoustic therapy purposes. In addition, the volume limit was established in the mobile device according to the audiometry curve of each patient.

### Procedure

#### Study design

Patients of the National Institute of Rehabilitation suffering from refractory and chronic tinnitus, who had been received homeopathic treatments with no positive effects, are asked to take part in the present project. They are informed about the experimental procedure, and notified that their head physician is also following-up the project. We aim at recruiting around 60 patients in total, so as to have 15 patients per group.

**Fig. 9:**
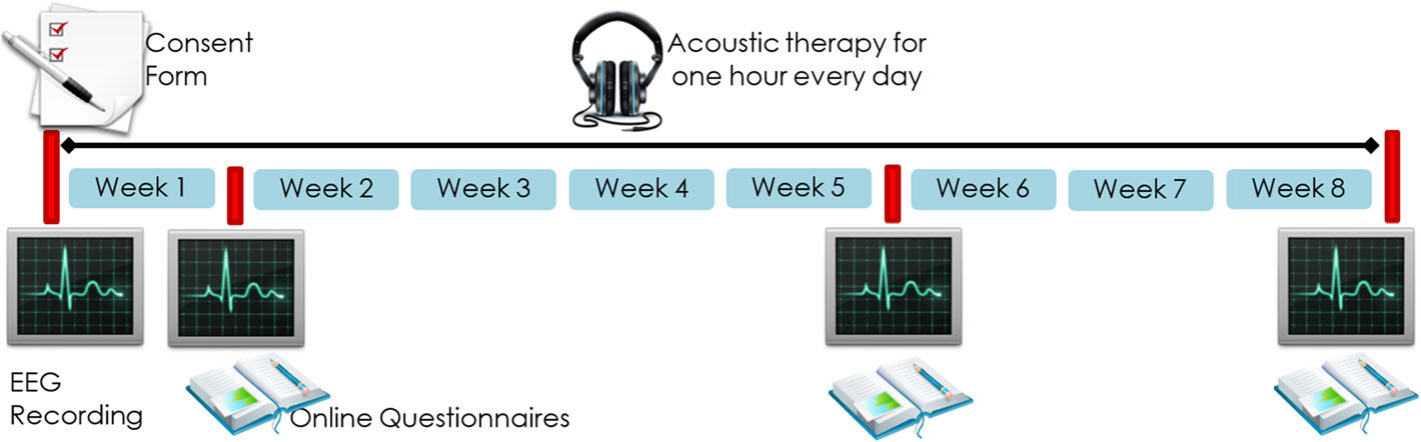
Study design of the protocol. Acoustic therapies are monitored for eight weeks. EEG signals are recorded at the beginning of the study, at one week of treatment, at five weeks of treatment, and at eight weeks of treatment. Once patients have completed the first week of treatment, they are required to fill in online questionnaires concerning the psychoacoustic evaluation

Once patients have been agreed to participate and a written consent form has been obtained, they are randomly assigned into one of five groups: control, ADT, TRT, EAE, and binaural [97]. They are instructed to use the assigned therapy for one hour, every day, and at any time of the day. The therapy is monitored for eight weeks as is depicted in Fig. 9. During that span, four EEG recordings and three online questionnaires are conducted. The first EEG recording is taken at the beginning of the study. The other three recordings are respectively carried out one, five and eight weeks after therapy initialization. EEG data is recorded in four different conditions: rest, acoustic therapy, passive mode and active mode. Each EEG session lasts around 60 min and has been organized as shown in Table 1. As can been seen from the table, resting condition (Section Resting Condition) is recorded in session 1, acoustic therapy condition (Section Acoustic Therapy) is recorded in session 4, passive mode condition (Section Passive Mode) is taken in the four sessions, and active mode condition (Section Active Mode) is taken from second session to ahead. With regard to online questionnaires, these are filled in at three different spans: one, five and eight weeks after treatment initialization [98]. These questionnaires are intended as a psychological and subjective measurement to assess the emotional and behavioral irregularities related to tinnitus. The psychological part is based on the Hospital Anxiety and Depression Scale (HADS) presented in [99]. The tinnitus assessment is an ad-hoc version of the Tinnitus Handicap Inventory adapted from the National Institute of Rehabilitation, and created originally in [100]. As questionnaires are a psychological measurement that can reveal emotional and behavioral irregularities related to tinnitus, they could be later associated with EEG outcomes.

**Table 1:** Organization of the experimental procedure

		*Total Duration per Session*			
		*Session 1*	*Session 2*	*Session 3*	*Session 4*
		*39 min*	*53 min*	*53 min*	*56 min*
Duration per Activity	15 min	Consent form			
	15 min		Online questionnaire		
	15 min	Electrode mounting			
	6 min	EO/EC			
	3 min				Therapy
	3 min	Passive mode			
	20 min		Restaurant	Park	Construction in progress

##### Resting condition

Resting condition is recorded in session 1; that is, at the beginning of the study when no therapy has been applied yet. Patients are asked to sit down on a comfortable chair, to be relax, and to keep their eyes open (EO) for three minutes. Their vision is directed to a fixation point on the computer screen in use. After EO period, they are asked to keep their eyes close (EC) for the following three minutes. Although EO and EC conditions have been the most examined brain state in neuroimaging studies related to tinnitus perception, no agreement on which condition is most reasonable, or what duration is more convenient, has been reached yet. Either EO or EC, and duration variation from two [65] up to ten minutes [60] have been reported in previous studies.

In the present protocol, we propose to record periods of three minutes, in line with prior research conducted to determine neurophysiological parameters that depend on mental states at rest such as level of attention [101], alpha peak frequency [102], and level of motor imagery ability [103]. With respect to the selection between EO and EC, and even though both conditions are recorded at this stage, EC seems to be more reasonable to evaluate the tinnitus perception in the rest of the experimental stages (acoustic therapy, passive mode, and active mode). In [77], it was reported that enhanced gamma activity, which had been previously related to tinnitus perception, most probably proceeded from involuntary eye movements and saccades, rather than abnormal neural activity due to tinnitus. On the other hand, it was associated eye closure with diminution of high frequency brain oscillations in [104]. Taken together, these results suggest that if gamma ERS exists owing to tinnitus perception, such neural synchronization should be more detectable in EC, than in EO condition. In addition to this evidence, it was argued that alpha band rhythms can work as biomarker to differentiate brain states that require different attentional degrees such as EC and EO [101]. As EO involves interaction with the environment (external attentional process), and tinnitus perception is an internal attentional process, EC might be a more effective condition to study the effect of acoustic therapies during the auditory retraining.

##### Acoustic therapy

This condition is recorded in last session (fourth session), when the assigned acoustic therapy has been already applied for two months. Similar to previous condition, patients are asked to keep their EC for three minutes while they listen to their acoustic therapies. As was aforementioned, this condition (acoustic therapy) and the rest of them (passive and active modes) are only monitored in EC mode.

EEG mapping of tinnitus perception during acoustic therapy might reveal if there is any neural synchrony modification as a result of the treatment, in comparison with resting condition when this had not been received yet. In this way, if any beneficial effects have been produced during acoustic retraining, these should be reflected in this particular recording. An important point to remark is that the main goal of the project is to evaluate the therapy effect (independent variable), rather than tinnitus perception per se. Consequently, acoustic stimulation always depends on the acoustic therapy in use.

##### Passive mode

Passive mode is the only condition that is recorded in all the sessions: from session 1 to session 4. As the preceding condition, EEG recordings are only made with EC. In passive mode, auditory ERPs are examined, and for that purpose, stimulus with duration of 1 s and an inter-stimulus interval of 2.5 s are used. In total, 50 trials are taken, and hence each session lasts around two minutes.

Auditory ERPs have been extensively employed to study the tinnitus nature, and generally, 1 kHz tones have been preferred because of convenience (optimum responses over the auditory cortex), rather than for relevance. In other investigations, tinnitus sound has been emulated, and subsequently, it has been used as auditory stimulus [77]. However, it is important to keep in mind that tinnitus frequency response curve is subjectively obtained in humans [14], and we would depend on patients’ perception to establish auditory stimulus, what in turn could produce subjective outcomes. The use of auditory stimulus based on the acoustic therapy at hand would standardize the evoke response, at least in each group. Acoustic therapies are moreover the subject of the study, and it seems plausible to provide the same auditory stimulation used to achieve acoustic retraining, as well as to assess such retraining.

##### Active mode

Active mode is recorded in sessions 2, 3, and 4, and EC is requested as well. In each session, a usual acoustic environment is played, whilst five associated auditory stimuli are randomly played. Patients are instructed to identify the randomized stimuli by pressing a keyboard button. The acoustic environments along with their related stimuli in each session are the following: (1) *restaurant* – soda can being opened, door closing, glass breaking, microwave sound, and human sound (tasting food); (2) *park* – camera clicking, book page turning, cards shuffling, human sound (laughing), and human sound (whistling); and (3) *construction in progress* – hit, bang, mobile dialing, police siren, and human sound (yelling). All the stimuli have been standardized to 1 s and are repeated 50 times at a random rate [105].

Although tinnitus perception has been related to abnormal neural activity in the auditory system, tinnitus distress has been associated with co-activation of frontal, limbic, memory and automatic systems [48, 77]. Memory is of particular interest because it is a sensory representation of the world, which allows humans interacting with their environment. In essence, new input patterns are first decoded and storage in the long-term memory of the brain. When those patterns appear again, brain retrieves information from memory, and makes a prediction. In this way, the consumption of mental resources is reduced [106]. What we pursue in active mode registry is to investigate if patients suffering from chronic tinnitus have memory atrophies, by evaluating their reaction time to recognize common sounds in typical environments. This EEG evaluation might be a way to assess indirectly tinnitus distress.

At present, no reliable EEG-based methods have been found. In fact, some authors [60] consider that EEG is not an effective way to study tinnitus effects. On this evidence, several EEG evaluations have been proposed in this protocol: from no specific auditory stimulation (resting condition), to auditory pattern recognition (active mode). Possibly, this set-up could also lead us to propose an efficient EEG-based methodology to study tinnitus effects.

#### Selection criteria

A random sample of around 60 patients without any history of otitis, cerebellopontine angle tumors, psychiatrist pathologies, demyelinating diseases of the nervous system, or epilepsy is expected to be recruited. Patients can be either female or male, they must be older than 18 years old, they must accept voluntarily to participate in the project, and they must sign a consent form. Owing to the expected difficulty in obtaining participants, patients with normal audition, unilateral or bilateral hyperacusis, and/or conductive sensory-neural hyperacusis are included. It is worth noting that the National Institute of Rehabilitation provides the following information about each patient: *tinnitus sound matching* (i.e., perception of tinnitus), *minimum masking level* (i.e., volume at which an external narrowband noise masks or covers), and *loudness discomfort level* (i.e., volume at which external sound becomes uncomfortable or painful for a tinnitus patient).

#### Post-study treatment

In the case that patients do not report either habituation or suppression to tinnitus during the two-month treatment, standard versions of acoustic therapies are provided to patients, if they decide to continue with the post-study treatment. Only four of the five therapies in use are applied in post-study treatment because the one originally assigned to the patient must be discarded. Patients are instructed to use each therapy every day for 30 min and during two weeks. After this testing period, patients must report if they detect any positive change. If any, the therapy of interest is adjusted to the patient audiometry. For the post-study treatment stage, no EEG monitoring is undertaken since it is beyond the scope of the project.

## Discussion

A limitation of this research proposal is the guarantee of acoustic therapy application as is indicated. This procedure cannot be verified as drug administration can be via blood test. In order to increase reliability, only liable patients are recruited according to a previous filtering process undertaken in the National Institute of Rehabilitation. Furthermore, patients are notified about the experimental procedure, and they must sign a compromise statement, agreeing to take part in the program. As a further work, an application in a mobile device can be implemented to measure the exposition time to the therapy.

Another limitation of the proposal is the number of EEG channels. As can be seen from Fig. 7, only 17 of the 19 electrodes of the 10/20 system are mounted. However, we consider that the small number of available channels may restrict somehow the study, but the estimation of EEG measures is still feasible and reliable. It is well-known that the spatial resolution of EEG is very poor and underdetermined, even if a large number of sensors are used. EEG signals are smeared as they pass through the surrounding tissue and the poorly conductive skull. Furthermore, the surface distribution of electrical currents is also somewhat distorted since the conductivity and the thickness of the skull is non-uniform [77]. The determination of signal sources is, therefore, complex and inaccurate due to EEG nature, rather than the number of sensors in use. Up to now, data acquisition in previously conducted EEG-based studies has been made using from 19 [64] to 128 [5] sensors. As can been seen, consensus has not been reached yet, and even the use of too many channels does not guarantee the analysis of all of them. For instance, in [5], it was reduced the number of electrodes from 128 to 109 channels by omitting the outermost ring of electrodes, as they usually show high amounts of noise. Lastly, the study of certain EEG measures such as ERPs is commonly undertaken only over one or two recording sites, what is achievable by means of the EEG layout depicted in Fig. 6. In conclusion, we consider that recordings of 17 EEG channels can be exploited to obtain consistent results.

## Abbreviations

ADT: Auditory Discrimination Therapy
EAE: Enriched Acoustic Environment
EC: Eyes-close
EEG: Electroencephalography
EO: Eyes-open
ERD: Event-related Desynchronization
ERO: Event-related Oscillations
ERP: Event-related Potentials
ERS: Event-related Synchronization
TRT: Tinnitus Retraining Therapy
